# Designed hybrid TPR peptide targeting Hsp90 as a novel anticancer agent

**DOI:** 10.1186/1479-5876-9-8

**Published:** 2011-01-14

**Authors:** Tomohisa Horibe, Masayuki Kohno, Mari Haramoto, Koji Ohara, Koji Kawakami

**Affiliations:** 1 Department of Pharmacoepidemiology, Graduate School of Medicine and Public Health, Kyoto University, Yoshida Konoecho, Sakyo-ku, Kyoto, 606-8501, Japan

## Abstract

**Background:**

Despite an ever-improving understanding of the molecular biology of cancer, the treatment of most cancers has not changed dramatically in the past three decades and drugs that do not discriminate between tumor cells and normal tissues remain the mainstays of anticancer therapy. Since Hsp90 is typically involved in cell proliferation and survival, this is thought to play a key role in cancer, and Hsp90 has attracted considerable interest in recent years as a potential therapeutic target.

**Methods:**

We focused on the interaction of Hsp90 with its cofactor protein p60/Hop, and engineered a cell-permeable peptidomimetic, termed "hybrid Antp-TPR peptide", modeled on the binding interface between the molecular chaperone Hsp90 and the TPR2A domain of Hop.

**Results:**

It was demonstrated that this designed hybrid Antp-TPR peptide inhibited the interaction of Hsp90 with the TPR2A domain, inducing cell death of breast, pancreatic, renal, lung, prostate, and gastric cancer cell lines *in vitro*. In contrast, Antp-TPR peptide did not affect the viability of normal cells. Moreover, analysis *in vivo *revealed that Antp-TPR peptide displayed a significant antitumor activity in a xenograft model of human pancreatic cancer in mice.

**Conclusion:**

These results indicate that Antp-TPR peptide would provide a potent and selective anticancer therapy to cancer patients.

## Background

Heat-shock protein 90 (Hsp90) is a molecular chaperone [1] that participates in the quality control of protein folding. The mechanism of action of Hsp90 includes sequential ATPase cycles and the stepwise recruitment of cochaperones, including Hsp70, CDC37, p60/Hsp-organizing protein (Hop), and p23 [2,3]. In particular, Hsp90 and Hsp70 interact with numerous cofactors containing so-called tetratricopeptide repeat (TPR) domains. TPR domains are composed of loosely conserved 34-amino acid sequence motifs that are repeated between one and 16 times per domain. Originally identified in components of the anaphase-promoting complex [4,5], TPR domains are now known to mediate specific protein interactions in numerous cellular contexts [6-8]. Moreover, apart from serving mere anchoring functions, TPR domains of the chaperone cofactors Hip and p60/Hop also are able to regulate the ATPase activities of Hsp70 and Hsp90, respectively [9,10]. Each 34-amino acid motif forms a pair of antiparallel α-helices. These motifs are arranged in a tandem array into a superhelical structure that encloses a central groove. The TPR-domain-containing cofactors of the Hsp70/Hsp90 multi-chaperone system interact with the C-terminal domains of Hsp70 and Hsp90 [11]. Studies involving deletion mutagenesis have suggested that the C-terminal sequence motif EEVD-COOH, which is highly conserved in all Hsp70s and Hsp90s of the eukaryotic cytosol, has an important role in TPR-mediated cofactor binding [12]. Hop serves as an adapter protein for Hsp70 and Hsp90 [13,14], optimizing their functional cooperation [15] without itself acting as a molecular chaperone [16], and contains three TPR domains, each comprising three TPR motifs [17]. The N-terminal TPR domain of Hop, TPR1, specifically recognizes the C-terminal seven amino acids of Hsp70 (PTIEEVD), whereas TPR2A recognizes the C-terminal five residues of Hsp90 (MEEVD) [17].

Hsp90 has a restricted repertoire of client proteins; for example, several kinases, among other proteins, that bind to Hsp90 for proper maturation, and Hsp90 is typically involved in cell proliferation and survival [2,3]. This is thought to play a key role in cancer [18-20], in which the stress-response recognition of Hsp90 may help promote tumor-cell adaptation in unfavorable environments [21]. Understanding of this pathway has created a viable therapeutic opportunity [22], and molecular targeting of Hsp90 ATPase activity by the class of ansamycin antibiotics prototypically exemplified by geldanamycin [23] has shown promising anticancer activity by disabling multiple signaling networks required for tumor-cell maintenance [24]. Although many Hsp90-targeted compounds are being examined for anticancer therapeutic potential, the molecular mechanism of their anticancer activity is still unclear. Recently, Gyurkocza *et al*. reported a novel peptidyl antagonist of the interaction between Hsp90 and survivin, and designated it "shepherdin" [25,26]. Survivin is a member of the inhibitor of apoptosis gene family [27] and is involved in the control of mitosis and the suppression of apoptosis or cell death [28]. It is demonstrated that shepherdin makes extensive contacts with the ATP pocket of Hsp90, destabilizes its client proteins, and causes massive death of cancer cells by apoptotic and nonapoptotic mechanisms. Strikingly, shepherdin does not reduce the viability of normal cells [25,26]. These results indicate that not only small compounds but also peptides targeting Hsp90 would provide potent antitumor selectivity in a cancer-bearing host.

In this study we designed a novel hybrid peptide consisting of cell-membrane-penetrating and Hsp90-targeted sequences. Structure-based mimicry to disrupt the interaction between Hsp90 and the TPR2A domain of Hop was demonstrated, as were the efficacies *in vitro *and *in vivo *of this peptide drug against cancer.

## Methods

### Materials

Anti-Hsp90 and anti-Hsp70 antibodies, human recombinant Hsp90α, and Hop (p60) were purchased from Stressgen Bioreagents. Anti-Akt and anti-CDK4 antibodies were purchased from Cell Signaling. Anti-survivin antibody was purchased from Thermo Scientific. Human recombinant FKBP5 and PP5 were purchased from Abnova. Anti-β-actin antibody and human recombinant Hsp70 were purchased from SIGMA. All reagents were of reagent-grade quality.

### Strain and plasmid

*Escherichia coli *AD494 (DE3) {Δ*ara*, *leu *7697, Δ*lac*X74, Δ*pho*A, *Pvu*II, *Pho*R, Δ*mal*F3, F' [*lac*^-^, (*lacI*^*q*^), *pro*], *trx*B::*kan *(DE3)} and pET-15b (Novagen Inc.) were used for expression of the TPR2A domain of human Hop.

### Cell culture

The following human tumor and normal cell lines were obtained from the American Type Culture Collection (ATCC): human breast cancer (BT-20, T47D, and MDA-MB-231), lung cancer (A549), kidney cancer (Caki-1), prostate cancer (LNCap), gastric cancer (OE19) and lung fibroblast (MRC5). Human pancreatic cancer cell line (BXPC3) was purchased from the European Collection of Cell Culture (ECACC). Human embryonic kidney cell line (HEK293T) and human normal pancreatic epithelial (PE) cell line ACBRI 515 were purchased from RIKEN cell bank and DS Pharma Biomedical, respectively. Cells were cultured in RPMI-1640 (BT-20, MDA-MB-231, T47D, LNCap, OE19, and BXPC3), MEM (MRC5 and A549), DMEM (HEK293T and Caki-1) or CSC (PE) containing 10% fetal bovine serum (FBS), 100 μg/ml penicillin, and 100 μg/ml streptomycin.

### Peptide synthesis

Peptides used in this study were synthesized by Invitrogen or SIGMA. All peptides were synthesized by use of solid-phase chemistry, purified to homogeneity (i.e. >90% purity) by reversed-phase high-pressure liquid chromatography, and assessed by mass spectrometry. Peptides were dissolved in water and buffered to pH 7.4. The TPR sequence 301K-312K (KAYARIGNSYFK; TPR), TPR mutant 1 (KAYAAAGNSYFK; mutated amino acids are underlined), TPR mutant 2 (KAYARIGNSGGG), and scramble peptide (RKFSAAIGYNKY) were made cell-permeable by addition of helix III of the cell-penetrating Antennapedia homeodomain sequence (underlined below) [29], as follows: RQIKIWFQNRRMKWKKKAYARIGNSYFK (Antp-TPR), RQIKIWFQNRRMKWKKKAYAAAGNSYFK (Antp-TPR mutant 1), RQIKIWFQNRRMKWKKKAYARIGNSGGG (Antp-TPR mutant 2), and RQIKIWFQNRRMKWKKRKFSAAIGYNKY (Antp-scramble).

### Expression and purification of the TPR2A domain of human Hop

The TPR2A domain (223K-352L) of human Hop was cloned in-frame into the *Xho*I and *Bam*HI sites of pET-15b for expression in *E. coli *AD494 (DE3), and purified using a nickel-chelating resin column as described previously [30]. To confirm the presence of purified protein, SDS/PAGE was performed according to the method of Laemmli [31].

### Surface plasmon resonance (SPR)

SPR experiments were performed with the Biacore biosensor 3000 system as described previously [30,32]. Human recombinant Hop, FKBP5, PP5, and purified TPR2A domain of Hop proteins were immobilized on the surface of CM5 sensor chips via *N*-hydroxysuccinimide and *N*-ethyl-*N*'-(dimethylaminopropyl)carbodiimide activation chemistry according to the manufacturer's instructions. Biotin-conjugated TPR peptide (biotin-TPR) was immobilized on the surface of streptavidin (SA) sensor chip. As the analyte, several concentrations of Hsp90 or Hsp70 were injected over the flow-cell at a flow rate 30 μl/min at 25°C. HBS-EP buffer (0.01 M Hepes/0.15 M NaCl/0.005% Tween 20/3 mM EDTA, pH 7.4) was used as a running buffer during the assay to inhibit non-specific binding. Data analysis was performed using BIA evaluation version 4.1 software. Competition experiments were performed by preincubating Hsp90 or Hsp70 with short, defined peptides or combinatorial peptide mixtures according to the method of Brinker *et al*. [30]. Briefly, protein/peptide mixtures were passed over the immobilized Hop, FKBP5, PP5 or TPR2A domain of Hop, and bindings of Hsp90 or Hsp70 to these proteins were followed. SPR signals obtained in the absence of competing peptides were used as a reference (100% binding) to normalize values obtained in the presence of peptides. For competition experiments involving defined peptides the concentration of TPR protein was kept constant, whereas the peptide concentration of the protein/peptide mixtures was increased systematically.

### Western blotting

Western-blot analyses were carried out as described previously [33]. Briefly, protein extracts were prepared from cells lysed with buffer containing 1% (v/v) Triton X-100, 0.1% (w/v) SDS, and 0.5% (w/v) sodium deoxycholate, separated by SDS/PAGE, and transferred to nitrocellulose filters. Quenched membranes were probed with antibodies and analyzed using enhanced chemiluminescence reagent (GE Healthcare) with an LAS-3000 LuminoImage analyzer (Fujifilm).

### Assay for cell viability

Cells were seeded on to 96-well plates at 2000-3000 cells/well and incubated with the test peptide. After incubation, an assay for cell viability was carried out using Living Cell Count Reagent SF (Nacalai Tesque) according to the manufacturer's protocol. Absorbance was measured at a wavelength of 450 nm using a 96-well microplate reader (GE Healthcare).

### Fluorescent microscopy

BXPC3 cells were plated in a glass-bottomed dish at 1 × 10^6 ^cells per ml of medium, and small aliquots of labeled-peptides, Antp-TPR-TAMRA-OH or TPR-TAMRA-OH (Invitrogen) (15 μl) were added directly into the dish at a final concentration of 10 μM. After 2 hr incubation, intracellular penetration of the peptides was visualized by an Olympus FV1000 confocal laser scanning microscope (Olympus).

### Flow cytometry assay

After incubation with or without Antp-TPR peptide, cells were collected and washed twice with PBS. Following this, the cell pellets were resuspended. Flow cytometry (Becton Dickinson) analysis was performed using the Annexin V-Fluorescein Staining Kit (Wako) or carboxyfluorescein FLICA caspase 3 & 7 assay kit (immunochemistry Technologies) according to the manufacturer's protocol. Data were analyzed using CellQuest Software.

### Antitumor activity of Antp-TPR peptide in tumor xenografts in vivo

Animal experiments were carried out in accordance with the guidelines of the Kyoto University School of Medicine. Cells of the pancreatic cancer cell line BXPC3 (5×10^6 ^cells), resuspended in 150 μl of PBS, were transplanted subcutaneously into the flank region of 7-9-week-old athymic nude mice weighing 17-21 g. When tumors reached around 50 mm^3 ^in volume, animals were randomized into three groups, and PBS (control) or Antp-TPR peptide (1 or 5 mg/kg) was injected intravenously (50 μl/injection) three times a week for a total of nine doses. Tumors were measured with a caliper, and the tumor volume (in mm^3^) was calculated using the following formula: length×width^2^×0.5. All values are expressed as the mean ± SD and statistical analysis was calculated by a one-way ANOVA with Dunnett test. Differences were considered to be significance at *P *< 0.05.

### Immunohistochemistry

Immunohistochemical staining was performed as described previously [34]. Briefly, BXPC3 tumor from animals treated either with saline or Antp-TPR peptide (5 mg/kg) intravenously were harvested at the end of treatment, and subsequently embedded in paraffin after fixation with 10% formaldehyde in PBS. After deparaffinized and hydrated, tumor sections were treated with antibodies, and then peroxidase activity was detected by incubation in 0.05% of 3, 3'-diaminobenzidine tetrachloride in PBS (pH7.2) containing 0.012% of H_2_O_2_.

## Results

### Design of TPR peptide

**Figure 1:**
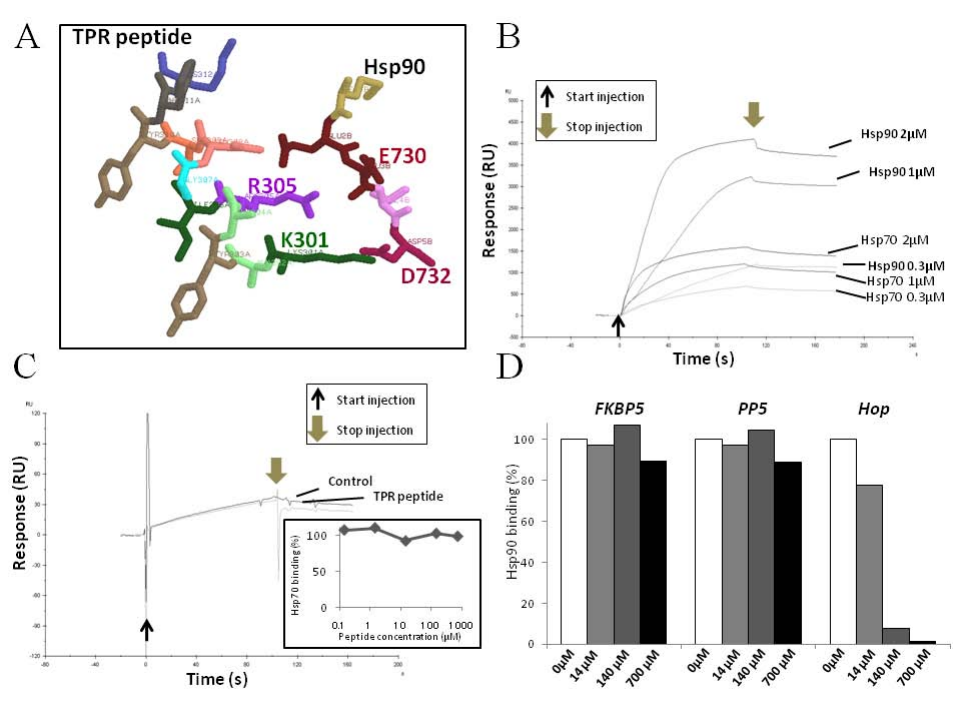
**Design and characterization of TPR peptide**. **(A) Predicted structure of designed TPR peptide**. The designed TPR peptide obtained from helix A3 of the TPR2A domain and the bound C-terminal region of Hsp90 are shown with stick model using Ras Mol software. Each number indicates the position of amino acids in Hop or Hsp90 proteins. (B) Sensorgrams of Hsp90 or Hsp70 bound to immobilized TPR peptide as determined using the Biacore biosensor. All analytes (0.3, 1, or 2 μM of Hsp90 or Hsp70) were injected over TPR peptide. The progress of binding to immobilized TPR peptide was monitored by following the increase in signal (response) induced by analytes. The thin and thick arrows indicate the start and stop injection, respectively. RU indicates resonance unit. (C) Competition assay for Hsp70 binding to Hop by TPR peptide. Hsp70 (1 μM) was passed over immobilized Hop in the absence (Control) or presence of TPR peptide (700 μM). The SPR signal in the absence of competing peptides was used as a reference (100% binding). Thin and thick arrows indicate start and stop injections, respectively. Equilibrium response levels obtained in the presence of competing peptides - TPR peptide was normalized and plotted against the peptide concentrations as described in the Materials and Methods section (inset graph). (D) Competition for Hsp90 binding to Hop, FKBP5, or PP5 with TPR peptide. Hsp90 (1 μM) was passed over immobilized Hop, FKBP5, or PP5 in the absence or presence of increasing concentrations of TPR peptide (14, 140, or 700 μM). The SPR signal in the absence of competing peptides was used as a reference (100% Hsp90 binding).

It is well known that the functional form of Hsp90 is a complex in which the chaperones Hsp90 and Hsp70 are brought together by binding to Hop [14] and assembly of this multiprotein complex is achieved by means of two independent TPR1 and TPR2A domains on Hop. In the complex of TPR2A and C-terminal region of Hsp90, Lys 301 and Arg 305 in helix A3 of TPR2A donate hydrogen bonds to the respective side chains of Asp and Glu of the Hsp90 C-terminal region [17]. In addition, Arg 305 in helix A3 is highly conserved among other TPR domains [17], and mutation of this Arg residue to Ala in the TPR domain of Tom70 is critical for binding to Hsp90 [35]. Based on these information, we designed a new TPR peptide, KAYARIGNSYFK, which includes the significant and highly conserved amino acids Lys 301 and Arg 305 for binding to Hsp90, using structural information obtained from the TPR2A-Hsp90 complex (Figure 1A). As shown in Figure 1(B), both Hsp90 and Hsp70 bind to the immobilized TPR peptide, and with similar *K*_*D *_values, 1.42 × 10^-6 ^(M) and 0.68 × 10^-6 ^(M) at increasing ligand concentrations, respectively, but the relative binding ability of Hsp70 to TPR peptide for Hsp90 was 49.9% (data not shown). In addition, the *K*_*D *_value of the interaction of Hsp90 with Hop was also similar (4.43 × 10^-6 ^(M), data not shown). It was found that the TPR peptide did not inhibit the interaction of Hsp70 with Hop protein as assessed by Biacore biosensor (Figure 1C), and that this peptide also did not affect the interaction of Hsp90 with FKBP5 or PP5 proteins (Figure 1D), which also have Hsp90-binding TPR domain as described previously [17]. However, it was shown that TPR peptide inhibited the interaction of Hsp90 with Hop protein (Figure 1D). The designated TPR peptide was further fused by its N-terminus to helix III of the Antennapedia homeodomain protein [29] to generate a cell-permeable variant, hybrid Antp-TPR peptide, as described in the Materials and Methods section.

### Selectivity of hybrid Antp-TPR and the significance of highly conserved amino acids in TPR peptide for anticancer activity

**Figure 2:**
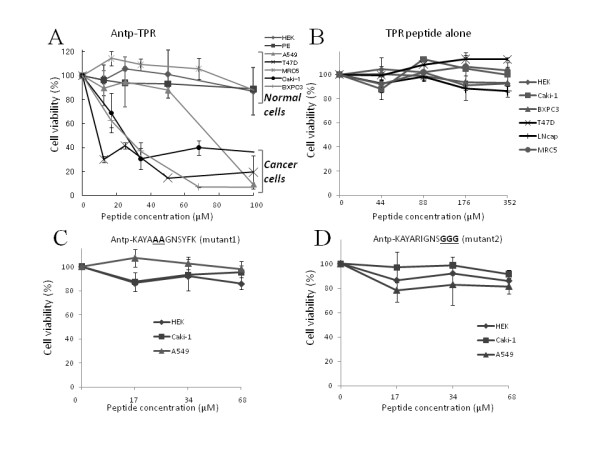
**Designed hybrid Antp-TPR peptide demonstrates selectivity for cancer-cell killing**. (A) The indicated cancer or normal cell lines were incubated with Antp-TPR peptide. (B) TPR peptide needs to be combined with Antp, the cell-penetrating peptide, to have a selective cell-killing effect. (C, D) Mutation analysis of TPR peptide examining its effect on cell killing. The indicated cell lines were incubated with Antp-TPR mutant 1 (C), in which highly conserved Arg and the subsequent amino acid, Ile, in the TPR peptide were replaced with Ala, or mutant 2 peptide (D), in which last three amino acids of TPR, Tyr-Phe-Lys, were replaced with three Gly residues to disrupt the helix structure of TPR. All cell viability was analyzed after 72 h incubation of test peptides as described Materials and Methods section. Data represent the mean ± SD from experiments performed in triplicate.

Based on analysis of the interaction of the designed hybrid Antp-TPR peptide with human Hsp90 protein, we then examined cancer-cell viability to assess the selectivity of this peptide in discriminating between normal and cancer cells. As shown in Figure 2(A), the Antp-TPR peptide caused a concentration-dependent loss of human cancer cell viability (in the Caki-1, BXPC3, T47D, and A549 cell lines); however, identical concentrations of this peptide did not apparently reduce the viability of normal human cell lines (HEK293T, MRC5, and PE) (Figure 2A), and TPR peptide without Antp, the cell-permeable peptide, had no effect on normal or cancer cells (Figure 2B). Confocal microscopy analysis also demonstrated that Antp-TPR peptide labeled with TAMRA penetrated the cancer cells, whereas TPR-TAMRA peptide without Antp sequence did not penetrate to cancer cells (Additional file 1). In addition, Antp-scramble peptide had no effect on these cell lines (data not shown). For the cancer cell lines tested, Antp-TPR peptide showed IC_50 _values of between 20 and 60 μM. On the other hand, TPR peptide showed no cytotoxicity towards either these cancer cell lines or normal cells (Table 1). These results demonstrate that the TPR peptide combined with Antp, a cell-permeable peptide, has selective anticancer activity that discriminates between normal and tumor cells. In addition, as shown in Figure 2(C) and 2(D), Antp-TPR mutant 1 and 2 peptides did not show selective antitumor activities when these peptides were tested with both normal and cancer cell lines. This suggests that the mutated amino acids in Antp-TPR mutants 1 and 2 are indispensable for the selective antitumor activity of Antp-TPR.

**Table 1:** Inhibitory concentration (IC_50_) of the Antp-TPR

Antitumor activity, IC_50 _(μM) *		
**Cell lines**	**TPR**	**Antp-TPR**

***Normal cells***		
HEK293T	-	>100
MRC5	-	>100
PE	-	>100
***Breast cancer***		
T47D	-	19.4
BT20	-	37.4
MDA-MB-231	-	56.9
***Pancreatic cancer***		
BXPC3	-	44.8
***Renal cancer***		
Caki-1	-	47.9
***Lung cancer***		
A549	-	65.9
***Prostate cancer***		
LNcap	-	56.7
***Gastric cancer***		
OE19	-	33.4

* Results are the mean of three independent experiments each performed in triplicate.- indicates no effect.

### Competition for TPR2A-mediated protein interactions by the designed TPR peptide

**Figure 3:**
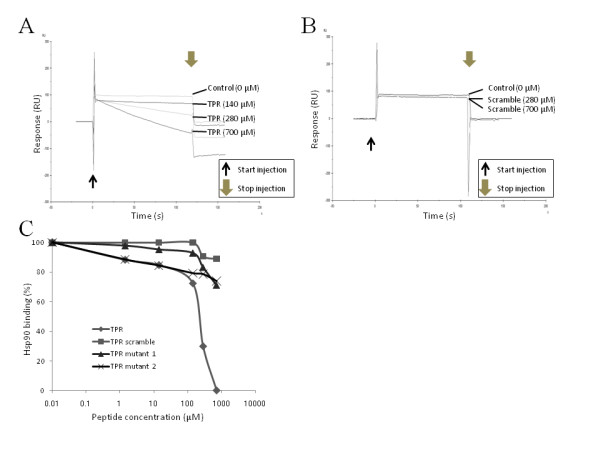
**Competition for Hsp90 binding to the TPR2A domain of Hop**. Hsp90 (1 μM) was passed over immobilized TPR2A domain of human Hop (5000 resonance units [RU]) in the absence or presence on increasing concentrations of TPR (A) or TPR scramble (B) peptide (1.4, 14, 140, 280, and 700 μM, and 1 mM). The SPR signal in the absence of competing peptides was used as a reference (100% binding). (C) Equilibrium response levels obtained in the presence of competing peptides - TPR (KAYARIGNSYFK), TPR scramble (RKFSAAIGYNKY), TPR mutant 1 (KAYAAAGNSYFK), or TPR mutant 2 (KAYARIGNSGGG) - were normalized and plotted against the peptide concentrations as described in the Materials and Methods section.

We further investigated whether the designed TPR peptide was able to compete specifically for the interaction of Hsp90 with the TPR2A domain of Hop, which is necessary for the correct folding of several oncogenic proteins in cancer cells [18-20]. Hsp90 was passed over a sensor chip carrying immobilized purified recombinant TPR2A domain of human Hop in either the absence or presence of increasing concentrations of TPR peptide (Figure 3). The SPR signal in the absence of peptide competitor was used as a reference (100% binding) to normalize the signals for Hsp90 binding recorded in the presence of peptide. The interaction of the TPR2A domain of Hop with Hsp90 was competed for by TPR peptide (Figure 3A and 3C). In contrast, the TPR scramble peptide and TPR mutant peptides 1 and 2 did not demonstrate any protein interaction when analyzed at up to millimolar concentrations (Figure 3B and 3C). These results indicate that the designed TPR peptide is a specific competitor capable of inhibiting the interaction between Hsp90 and the TPR2A domain of Hop, and that the amino acids targeted in our mutagenesis experiment are critical for this protein interaction to occur.

### Characterization of cancer cell killing and loss of client proteins by Antp-TPR peptide

**Figure 4:**
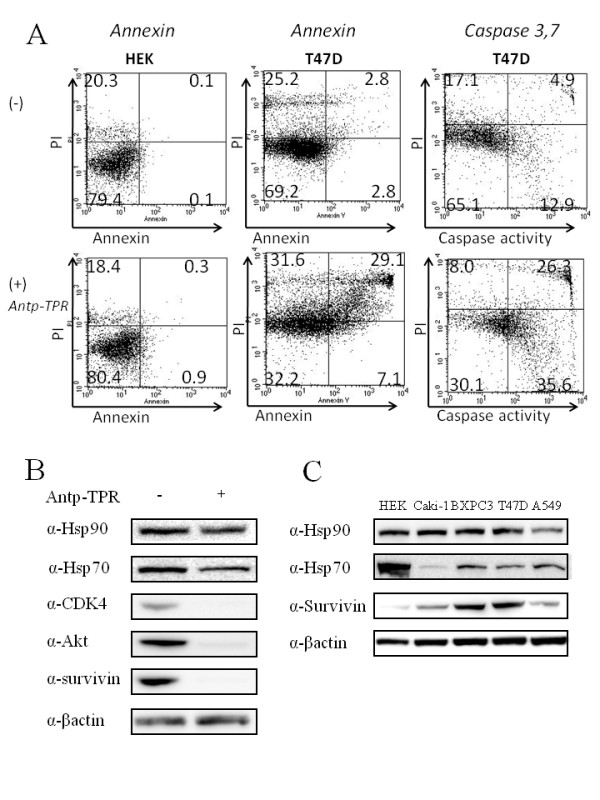
**Characterization of cancer cell killing and loss of client proteins by hybrid Antp-TPR peptide**. (A) HEK293T and T47D cells treated with (+) or without (-) Antp-TPR peptide (68 μM) were analyzed after 24 h by dual-color flow cytometry for annexin V (left and middle lane panels) or caspase 3 and 7 (right lane panels) labeling in the green channel, and propidium iodide (PI) staining in the red channel as described in the Materials and Methods section. The percentage of cells in each quadrant is indicated, and the experiments were performed twice with similar results. (B) Loss of Hsp90 client proteins. T47D cells were incubated with Antp-TPR peptide (68 μM) for 48 h and analyzed by Western blotting with the indicated antibodies. (C) Western-blot analysis of Hsp90, Hsp70, and survivin expression in the normal and cancer cell lines HEK293T, Caki-1, BXPC3, T47D, and A549. Cell extracts from these cell lines were examined for protein expression by Western-blot analysis. β-actin was used as the loading control.

As mentioned previously, the interaction of Hsp90 with Hop in cancer cells is significant for folding of several oncogene proteins including survivin, which is a member of the inhibitor of apoptosis gene family [27]. In addition, Antp-TPR has selective cytotoxic activity towards cancer cells and is an inhibitor of the interaction of Hsp90 with the TPR2A domain of Hop (Figures 2 and 3). These results prompted us to investigate whether Antp-TPR induces apoptosis in cancer cells. As assessed by flow cytometry analysis, annexin V or caspase 3 and 7 positive cells were found when Antp-TPR peptide was added to breast cancer T47D cells (Figure 4A, middle and right lane panels), suggesting that this peptide induces cancer cell death by apoptotic mechanism (Figure 4A, middle and right lane panels). On the other hand, there was no appearance of annexin V-labeled HEK293T cells after addition of this hybrid peptide (Figure 4A, left lane panels). Taken together with Figures 2 and 3, it was shown that the Antp-TPR peptide designed in this study provided selectivity to cancer cells, discriminating between normal and cancer cells.

When we examined the levels of Hsp90 client proteins after intracellular loading of Antp-TPR peptide, T47D cells treated with Antp-TPR exhibited loss of multiple Hsp90 client proteins, including survivin, CDK4, and Akt, as assessed by Western blotting (Figure 4B). In contrast, Antp-TPR peptide did not affect the levels of Hsp90 itself (Figure 4B). When normal and cancer cell lines (HEK293T, Caki-1, BXPC3, T47D, and A549) received heat shock, the up-regulation of Hsp90 and Hsp70 was observed in the cancer cells, but not in normal HEK293T cells (Additional file 2A). In addition, the up-regulation of Hsp70 after the treatment with this peptide was not observed in both cancer and normal cell lines (Additional file 2B). When we investigated the expression levels of Hsp90, Hsp70, and survivin in these cell lines using Western blotting, it was found that the expression of Hsp90 was almost equal between normal and cancer cells, however, survivin was highly expressed in cancer cell lines, and the expression level of Hsp70 was different among these cell lines (Figure 4C). These results suggest that the Antp-TPR peptide designed in this study would affect the cell-survival pathways in cancer cells by competing with cochaperone recruitment, which is indispensable for the correct folding of Hsp90 client proteins.

### Antitumor activity of Antp-TPR peptide in vivo

**Figure 5:**
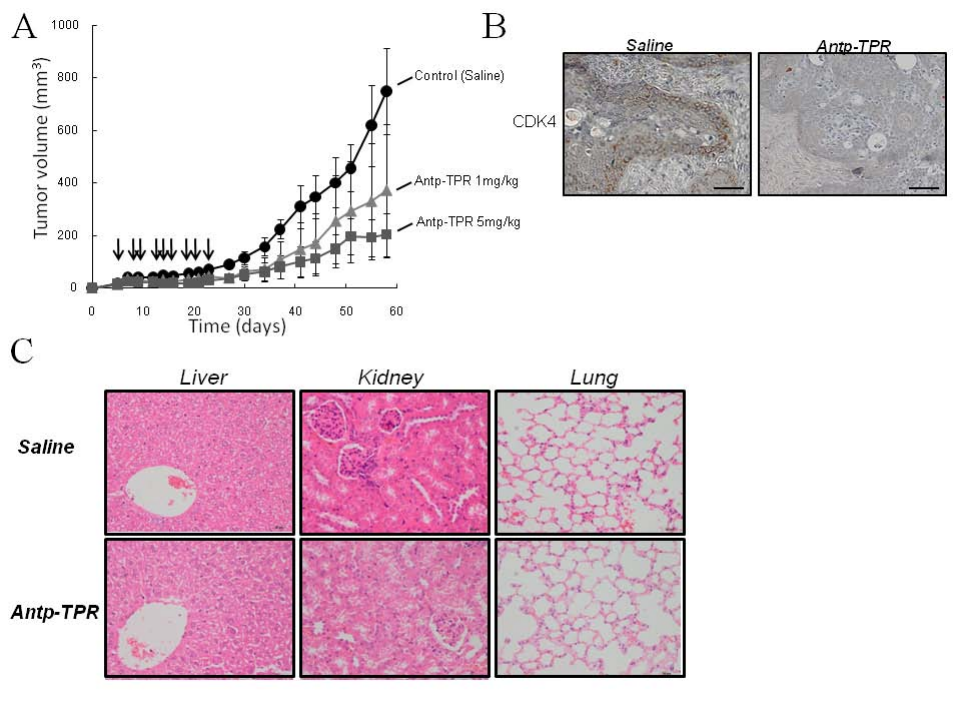
**Antitumor activity of hybrid Antp-TPR peptide *in vivo***. (A) BXPC3 pancreatic cancer cells were implanted subcutaneously into athymic nude mice. Intravenous injection of either PBS (control) or Antp-TPR peptide (1 or 5 mg/kg) was provided from day 4 as indicated by the arrows. Each group had six animals (n = 6), and experiments were repeated twice. Data are expressed as mean ± SD. (B) Loss of Hsp90 client protein (CDK4) in tumors treated with Antp-TPR peptide *in vivo*. BXPC3 tumors from saline or Antp-TPR peptide (5 mg/kg) treated animals were harvested at the end of treatment and analyzed with antibody to CDK4 by immunohistochemistry. Scale bars, 25 μm. (C) Histologic examination after treatment with Antp-TPR hybrid peptide. Images (x400 magnification) of liver, kidney, and lung from mice after treatment with saline (control), or Antp-TPR peptide (5 mg/kg) nine times were obtained by staining with hematoxylin and eosin (H & E).

To assess the antitumor effect of Antp-TPR peptide in a xenograft model of human cancer, BXPC3 pancreatic cancer cells were implanted subcutaneously into athymic nude mice and the animals were treated with Antp-TPR peptide. The control group exhibited progressive tumor growth, reaching 749 mm^3 ^at day 58 (Figure 5A). On the other hand, administration of Antp-TPR peptide (1 or 5 mg/kg, administered intravenously three times a week for 3 weeks) suppressed tumor growth remarkably. On day 58, mean tumor volume was 371 mm^3 ^in 1 mg/kg dosage group and 204 mm^3 ^in 5 mg/kg dosage group (*P *< 0.05 compared with control group) (Figure 5A). Immunohistochemical staining also demonstrated that Antp-TPR peptide caused loss of Hsp90 client protein (CDK4) in BXPC3 tumors *in vivo *after the treatment, although tumors from the saline group exhibited extensive labeling for this protein (Figure 5B). In addition, histologic examination of liver, kidney, and lung was equally unremarkable in the saline or hybrid peptide-treated mice (Figure 5C). These results suggest that the newly designed hybrid Antp-TPR peptide successfully induces tumor death via loss of Hsp90 client proteins *in vivo*.

## Discussion

In this study, we designed, identified, and characterized TPR peptide, a novel anticancer peptidomimetic modeled on the binding interface between Hsp90 and the TPR2A domain of Hop. As demonstrated in a recent structure-based approach, TPR2A discriminates between the C-terminal five residues of Hsp90 (MEEVD) and the C-terminal sequence of Hsp70 (PTIEEVD) with its main six helices (A1, B1, A2, B2, A3, and B3) [17,30]. In these helices, Lys 301 and Arg 305 of helix A3 are especially critical for their respective interaction by hydrogen bonding with the side chains of the Asp and Glu residues of the Hsp90 C-terminal peptide [17]. This information prompted us to design a peptide using the TPR2A domain of Hop, including the highly conserved Arg 305 residue of helix A3, that could compete for interaction with Hsp90, and to test the cytotoxicity of this peptide *in vitro *and its antitumor activity *in vivo*. Interestingly, both Hsp90 and Hsp70 were able to bind the designed TPR peptide (Figure 1B), however, the relative binding ability of Hsp70 to this peptide was lower than that of Hsp90, and this peptide failed to inhibit the interaction of Hsp70 with Hop protein (Figure 1C) and the interaction of Hsp90 with FKBP5 or PP5 (Figure 1D). In addition, TPR peptide inhibited the interaction of Hsp90 with Hop specifically. These results suggest that the designed peptide in this study is specific inhibitor to the interaction of Hsp90 with Hop protein. As shown in Figure (2A and 2B) and Additional file 1, the designed hybrid Antp-TPR peptide, with its cell-permeable sequence derived from the Antennapedia homeodomain, demonstrated selective antitumor activity, discriminating between normal and cancer cells. It was also demonstrated that mutating the TPR peptide by replacing the highly conserved Arg residue and the subsequent Ile in TPR2A helix A3 with double Ala (mutant 1) caused it to lose both its ability to inhibit the Hsp90-TPR2A interaction and its antitumor activity (Figures 2B, C, and 3). Another TPR peptide mutation, in which Tyr-Phe-Lys was replaced with triple Gly to disrupt the helical structure (mutant 2), turned out to have an effect similar to that of mutant 1, suggesting that these amino acids are critical for both inhibition and antitumor activities.

Interestingly, Antp-TPR peptide was cancer cell-specific in its cytotoxic activities and less cytotoxic to normal cells including HEK293T, PE, and MRC5 (Figure 2A), although the expression levels of Hsp90 did not differ very much between normal and cancer cells (Figure 4C). In contrast, survivin was expressed high in cancer cells (Figure 4C), and the sensitivity of these cancer cell lines to Antp-TPR correlated with the expression of this protein (Figure 2A). It is well-known that anti-apoptotic proteins such as survivin are over expressed in cancer cells, have significant roles for the suppression of apoptosis or cell death, and knockdown of these proteins in cancer cells sensitize to apoptosis [27,28]. Since cancer cells treated with this hybrid peptide were annexin V and caspase 3, 7 positive as assessed by flow cytometry, and this peptide also caused the loss of Hsp90 client proteins including survivin (Figure 4), we propose the mechanism of action of Antp-TPR peptide cancer cells killing as follows. First, Antp-TPR peptide inhibits the Hsp90-Hop interaction, and this inhibition affects the correct folding of these Hsp90 client protein including anti-apoptotic proteins such as survivin, and this effect might be critical especially in cancer cells to cause cell death by apoptotic mechanism. In addition, it was also found that Antp-TPR peptide did not cause up-regulation of Hsp70 after treatment with this peptide (Additional file 2B). Therefore it is suggested that this peptide might provide an additional advantage compared with Hsp90-targeted small compounds, since conventional Hsp90 ATPase inhibitors induce a compensatory up-regulation of Hsp70 that likely correlates with the decrease of anticancer activity as previously reported [36,37]. It was also demonstrated that Antp-TPR peptide had a significant antitumor activity in mice xenografted with human pancreatic cancer (BXPC3) causing loss of CDK4, which is one of Hsp90 client proteins in tumors. (Figure 5A and 5B), suggesting that this hybrid peptide administrated intravenously penetrates the tumor cells, inhibits the interaction of Hsp90 with Hop interaction, causes the loss of Hsp90 client proteins, and induces anti-tumor activity in vivo with similar mechanism shown in in vitro analysis. Moreover, histologic examination suggested that the administrated Antp-TPR peptide did not cause serious damages to the main organs (liver, kidney, and lung) and normal tissues, and any abnormal behaviors or losing of appetite after the treatment with this peptide was not also observed. These results suggest that this peptide may not cause serious side effect after the treatment. Taken together, these features of the designed Antp-TPR peptide would offer an attractive new anticancer therapeutic option for molecular targeted cancer therapy.

Previously we reported the antitumor activities of immunotoxins, comprising a targeting moiety, such as a ligand or an antibody to ensure cancer cell selectivity, and a killing moiety, such as a protein toxin [38-40]. These conventional immunotoxins usually present hurdles during clinical use, such as immunogenicity, undesirable toxicity, difficulty in manufacturing, limited half-life, and production of neutralizing antibodies [41-43]. However, chemical synthesis enables us to produce peptides affordably, with a cost comparable to that of producing protein drugs. Moreover, because of the easy production of peptides, a wide variety of candidate peptides combining moieties for targeting and toxicity can be tested in preclinical settings.

Recently, Gyurkocza *et al*. reported a novel peptidyl antagonist of the interaction between Hsp90 and survivin and demonstrated that this peptide causes massive death of cancer cells but does not reduce the viability of normal cells [25,26]. In addition, it was also reported that designed novel TPR modules, which binds to the C-terminus of Hsp90 with high affinity, decreased HER2 levels in BT474 HER2-positive breast cancer cells, resulting in the killing of these cells [44]. Taken together with our current study, these results indicate that peptides targeted at Hsp90 could be potent and novel selective anticancer agents.

## Conclusion

The newly designed hybrid Antp-TPR peptide described in this study has the molecular features of an inhibitor of Hsp90-Hop interaction, which is critical for the folding of several client proteins in cancer cells. Moreover, the analysis of this peptide *in vivo *revealed that it displays significant tumor-suppression activity in mice with human pancreatic tumor. Because of these features, Antp-TPR peptide may provide a potent and selective new cancer therapy, consistent with the use of peptidomimetics in targeted cancer therapy [45]. The findings of this study will assist the further elucidation of cancer treatment targeting Hsp90.

## Abbreviations

Hsp90: heat shock protein 90; Hop: p60/Hsp-organizing protein; TPR: tetratricopeptide repeat; Antp: antennapedia homeodomain sequence; IC50: the peptide concentration inducing 50% inhibition of control cell growth; PI: propidium iodide; SPR: surface plasmon resonance; RU: resonance unit; SDS: sodium dodecyl sulfate.

## Competing interests

Koji Kawakami is a founder and stock holder of Upstream Infinity, Inc. The other authors disclose no potential conflicts of interest.

## Authors' contributions

TH, MK, and KK designed this research work. TH desgined the Antp-TPR peptide, and performed binding, inhibition assay by SPR technique, and cell viability assay in vitro. KO performed the mechanism of cancer cells death by FACS analysis in vitro. MH and KO performed in vivo analysis by mouse xenograft model, KO carried out the immunocytochemistry analysis using tumor section after in vivo analysis. TH, MK, and KK, interpreted the data and wrote the manuscript. All authors read and approved the final manuscript.

## Supplementary Material

Additional file 1 **Intracellular penetration of antennapedia helix III homeodomain (Antp)-conjugated Antp-TPR hybrid peptide**. BXPC3 cells were incubated with 10 μM of carboxytetramethyl rhodamine (TAMRA)-labeled Antp-TPR (Antp-TPR-TAMRA) or TPR (TPR-TAMRA) as indicated. Cells were then analyzed by phase-contrast (DIC), fluorescence (TAMRA-red) or merge image (DIC and TAMRA-red). All images were taken using confocal laser scanning microscopy as described in Methods. All scale bars are 50 μm. Click here for file

Additional file 2 **Effect of heat shock or Antp-TPR peptide treatment on the expression levels of Hsp90 or Hsp70 protein in normal and cancer cells**. (A) Western-blot analysis in normal and cancer cell lines (HEK293T, Caki-1, BXPC3, T47D, and A549) receiving heat shock. Cell extracts after 2 hr of heat shock treatment (43°C) were examined for the expression of Hsp90 and Hsp70 by Western-blot analysis using specific antibodies. (B) Expression levels of Hsp70 in the normal and cancer cell lines (HEK293T, Caki-1, BXPC3, T47D, and A549) treated with hybrid Antp-TPR peptide. Cell extracts after treatment with Antp-TPR peptide were examined for the expression of Hsp70 by Western-blot analysis using specific antibodies. β-actin was used as the loading control. Click here for file
