# Supplementary figures and images for: Designed hybrid TPR peptide targeting Hsp90 as a novel anticancer agent
Source: J Transl Med. 2011 Jan 14;9:8. doi: 10.1186/1479-5876-9-8 (PMC3032679; doi:10.1186/1479-5876-9-8)

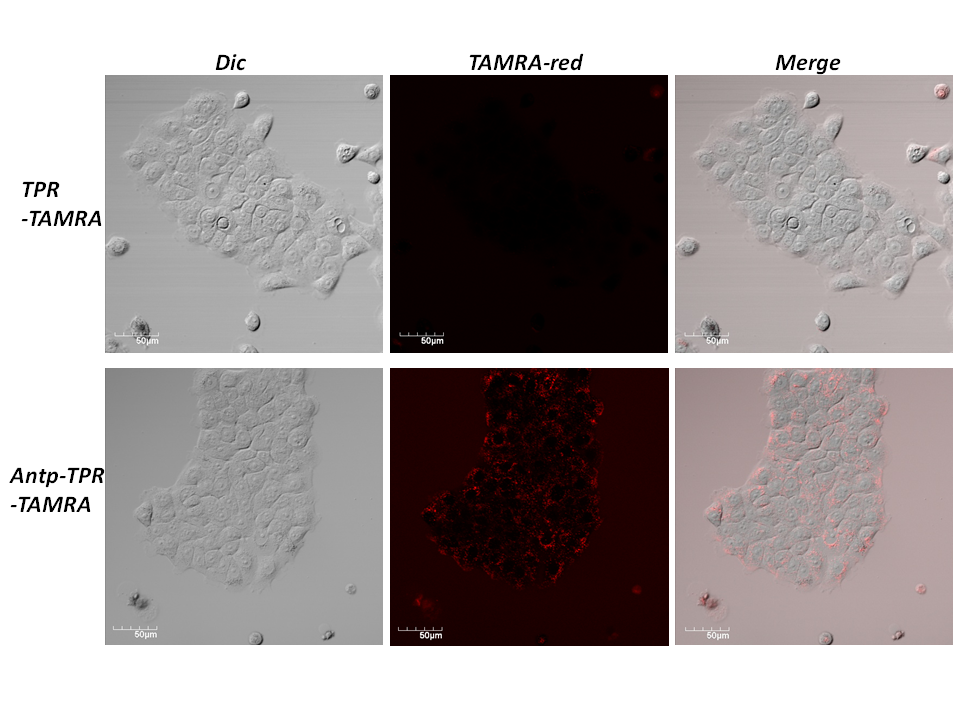

Supplement: Additional file 1 — Intracellular penetration of antennapedia helix III homeodomain (Antp)-conjugated Antp-TPR hybrid peptide. BXPC3 cells were incubated with 10 μM of carboxytetramethyl rhodamine (TAMRA)-labeled Antp-TPR (Antp-TPR-TAMRA) or TPR (TPR-TAMRA) as indicated. Cells were then analyzed by phase-contrast (DIC), fluorescence (TAMRA-red) or merge image (DIC and TAMRA-red). All images were taken using confocal laser scanning microscopy as described in Methods. All scale bars are 50 μm. [file 1479-5876-9-8-S1.TIFF]

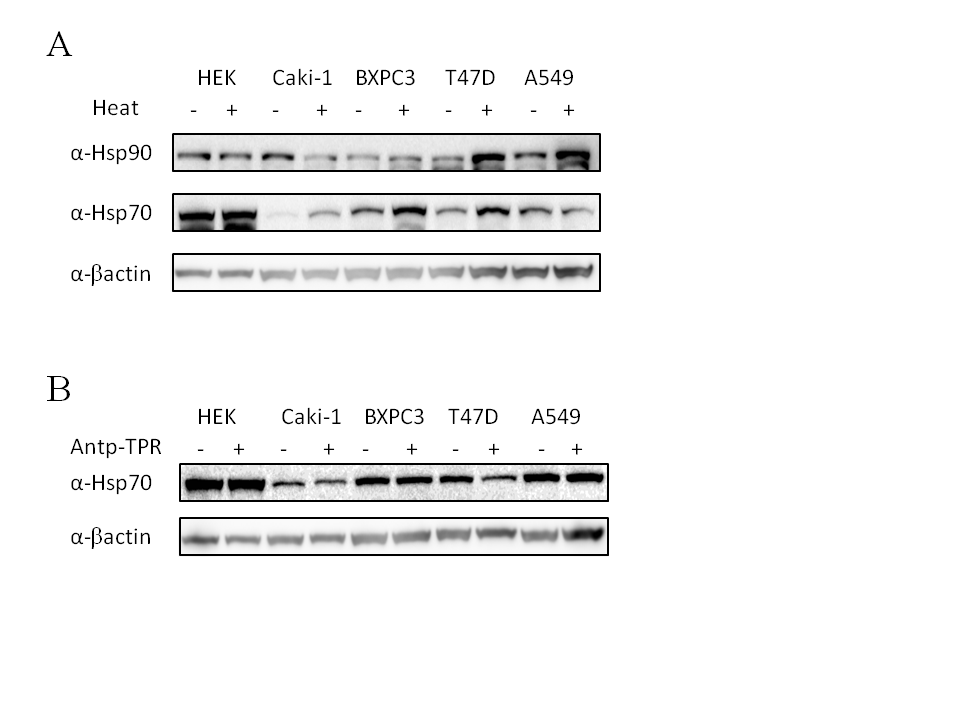

Supplement: Additional file 2 — Effect of heat shock or Antp-TPR peptide treatment on the expression levels of Hsp90 or Hsp70 protein in normal and cancer cells. (A) Western-blot analysis in normal and cancer cell lines (HEK293T, Caki-1, BXPC3, T47D, and A549) receiving heat shock. Cell extracts after 2 hr of heat shock treatment (43°C) were examined for the expression of Hsp90 and Hsp70 by Western-blot analysis using specific antibodies. (B) Expression levels of Hsp70 in the normal and cancer cell lines (HEK293T, Caki-1, BXPC3, T47D, and A549) treated with hybrid Antp-TPR peptide. Cell extracts after treatment with Antp-TPR peptide were examined for the expression of Hsp70 by Western-blot analysis using specific antibodies. β-actin was used as the loading control. [file 1479-5876-9-8-S2.TIFF]
